# A Case of Moral Insanity

**Published:** 1849-04

**Authors:** B. M. Wible

**Affiliations:** Louisville, Ky.


Art. III.—*A Case of Moral Insanity.* By B. M. Wible, M. D., of Louisville, Ky.

The subject of the following report, Robert M. Ewing, was 21 years of age, muscular, moderate embonpoint, about five feet ten inches high and well formed, had blue eyes, dark stiff hair disposed to stand erect, and sanguine complexion.

In 1841 he had an attack of convulsions, followed by coma, which persisted about thirty hours, from the effect of which he recovered slowly. As soon as his health was sufficiently restored he was sent to a school in the city, where he had been but a short time when he began to exhibit indications of violence of temper entirely new to his character. He would frequently become involved in difficulties with his school mates, which would often end in some act of violence, for which he would immediately manifest the greatest sorrow, in such manner as soon to pacify all concerned. A difficulty, however, occurred with his teacher which caused his removal from the school. It was remarked by his friends that, after his recovery from the attack above noticed, there was a change in the expression of his countenance, which began to wear a constant, fixed frown, whereas before the attack his countenance was remarkable for a frank, open expression. On being removed from the school he was placed in charge of a relative in the country, where he continued to manifest the same kind of temper, having repeated difficulties of a more or less violent character, all growing out of, or connected with, some imaginary slight or indignity by which his pride would be wounded. From this time he was sent from college to college, and from place to place, in the vain hope of finding some relief for his waywardness of character, and his morbid pride, which was so constantly the source of his difficulties. He had now become restless and fond of change, would soon tire of friends and places, seem happy on evry new change of residence and delighted with every new associate, but would in a very short time desire another change, it being impossible to keep him contented at any one place. From his situation in the country he was sent successively to St. Mary's, St. Joseph's, Hanover, Indiana, Lexington in Virginia, a private school in the mountains in Virginia, and the University of Virginia, with all of which he was at first delighted, but of each of which he soon tired, and left either without permission or in consequence of difficulty.

On leaving the University he went to Washington City, Philadelphia, New York, in each of which places he was found by friends of his father, who tried to induce him to return, but on getting money from them would become wild with excitement and forget his promises, until again found destitute, and was only brought home finally by a friend who kept him under observation until he came back. Before his return, however, his father consulted Mr. Wolfe, and Maj. Butler, in reference to the plan he should adopt with him, and expressed to them his fears that his mind was diseased, but no particular course was decided upon, and he was placed successively in the clerk's office of the county court, and of the chancery court, but he soon became disgusted with these pursuits, and was advised to undertake a journey with Mr. Bryant and other of his acquaintances to California, and proceeded as far as the eastern base of the Rocky Mountains, when he had difficulties with his associates, returned home, and disappointed the hope, that the trip would exert a salutary influence on his mind.

His father, still convinced that he was laboring under mental disease, again consulted his lawyers with a view of obtaining a writ of lunacy, to place him under moral and medical treatment, but his counsel advisedagainst it, fearing the effort would prove abortive, and as a consequence, he might imbibe hatred towards his father, and be driven to excesses greater than those of his previous life. After this he went to Russellville to study law with a relative, but in a short time got into numberless difficulties and returned home. Soon after this he became still more reckless and wayward, in consequence of a failure to win the affections of an estimable lady, to whom he had long been attached—and he determined, against the united wishes of his friends, to join the army. At the urgent solicitation of his father, however, he agreed to return to Russellville, and proceeded on his way as far as the mouth of Salt River, where he met Capt. Churchill recruiting a company of voltigeurs for the Mexican war. Without a moment's reflection, in accordance with his usual course of conduct he left the carriage, and joined the company as a common soldier. His father expostulated with him upon the folly of his course, but all expostulation was fruitless, in consequence of a delusive hope which, with him, amounted to thorough conviction, that he would soon be promoted to a high position and rank in the army. After arriving at Vera Cruz and joining his regiment, he was made sergeant-major, but the duties proved irksome to him, and becoming dissatisfied, he exchanged places with a private belonging to another company, again went into the ranks, and was in this position at the battles of Conteras and Cherebusco. During the armistice that followed, by the aid of friends, he employed a substitute, became a follower of the army, and remained with it during the subsequent battles. He staid in the City of Mexico after it was taken by the American army, five or six weeks, entered into various speculations, made several thousand dollars, but, with a prodigality peculiar to him, spent it all, and when he arrived at Vera Cruz was without a cent. His wayward conduct led the officers, with whom he associated, to look upon him as laboring under mental derangement. Mr. Vertrees, a member of the company to which he belonged, and the present representative in the Legislature from Hardin county, relates that, from near one of the gates around the City of Mexico, directly under the protection of the Mexican batteries whilst in action, he drove out three or four hundred mules and beef cattle to the camp of Gen. Twigg's division of the army; and the commanding officer witnessing this conduct, and forming a subsequent acquaintance, regarded him as laboring under mental disease. On his way home, at Vera Cruz, he got into the greatest difficulty, and returned to Louisville in March, 1848, and from that time until October, had many desperate affrays with his friends and associates, and finally, October 9th, had the unfortunate rencounter, for which he was confined in the Louisville jail,

His general health previous to his confinement in prison was usually good, and though he was extremely susceptible to external impressions, he possessed great power to resist them, but his mind appeared to be controlled entirely by surrounding influences. His face was generally flushed, the brows contracted, the eyes prominent and more or less injected, and he did not sleep, on an average, more than five or six hours during the twenty-four. He talked much of pecuniary matters, entertained various plans for making an immense estate, and seemed to believe that he would be worth hundreds of thousands of dollars, yet he was prodigal with money, and when he had it in possession seemed not to be contented until he wasted it. He drank occasionally but it was seldom to intoxication, and the effect of spirituous liquor upon him was variable; sometimes it would calm him, and at other times increase his natural excitement. Soon after his confinement in jail he became costive, and took strong cathartic medicines for a week before they acted; among them a dose of calomel, which ptyalized him, and he was troubled with spectral illusions, seeing cats sitting on his breast, bugs and flies on the wall or on his dress.

Dec. 2d. I visited him in the evening, found him excited, talkative, and supposed, from his manner, that he had been drinking, but was assured he had not since his confinement. During the following night he had several violent convulsions. Dr. J. W. Knight visited him, saw him in one of them, and at the time believed he would die immediately. He bled him twenty ounces, gave a drachm of laudanum, and there was no return of the convulsions during the night. I visited him next day, found him alarmed, frowning and starting when touched, pulse variable, but in the main full, compressible, and about 80. Had some headache, required cold wet cloths constantly to the scalp; noise from moving a chair or newspaper in the room distressed him, and he was apprehensive of a return of convulsions, and would not consent to be left alone a moment. During the following week most of the foregoing symptoms continued, and he remained restless, frequently changing his position in bed, was entirely sleepless, had frequent vomiting, and voided large quantities of colorless urine. Once he had retention of urine requiring the catheter, and at another time said that he felt like the convulsions were about to return. He took various cathartic medicines, among them, infusion of senna and rhubarb, which operated freely, and on Monday morning, a week after the attack of convulsions, he was found extremely prostrated and entirely blind.

He was now removed from jail to his father's residence; reaction soon took place, attended by some fever, and on visiting him the following day he remarked that he could see me, though indistinctly, and some few hours afterwards his vision was almost entirely restored. He was still sleepless, remained so during the night, and the next morning the convulsions returned with their former violence, and Dr. Knight again bled him twenty ounces. The next day Dr. H. M. Bullitt was called in consultation, and we found him in the following condition: lying in bed with a flushed face, contracted brow, giving the countenance the expression of an angry frown; eyes prominent, with a wild and more or less fixed stare; pupils contracted. His manner abrupt, and his speech hurried, though his answers to questions were given with apparent reluctance, as if afraid of making mistakes. He complained, when interrogated as to his feelings, of fixed uneasiness in the region of the occiput, and sense of tension through the temples. Said that yesterday the palm of his hand reflected his face like a mirror, but is conscious to-day that it was a spectral illusion. He remarked to me that I looked unnatural. His hearing is so acute that he is disturbed by the slightest noise, notices everything that is said or done in his room, and recognises instantly every one that enters. He has frequent spasmodic jerkings of the muscles of one limb only, but occasionally affecting the whole muscular system. Does not like to be approached and touched suddenly, and is disturbed by having his bed subjected to the slightest motion, any sudden impression exciting muscular twitchings. He has not slept for several nights; pulse 58; impulse of the heart very violent; surface uniformly warm, though he complains of heat about the head. Bowels inclined to be costive, though acted upon yesterday freely by cathartics; urine less high colored than natural, very copious, and voided without difficulty. Stomach somewhat irritable, and disposed to reject both food and medicines. Thirst somewhat urgent; says he has appetite, but his stomach retains nothing but black tea and toast.

Under the impression that there had been some manifest tendency to periodical exacerbations, quinine and opium were prescribed, with the three fold view of counteracting the periodical tendency, allaying the extreme excitability and muscular twitchings, and procuring sleep. In consequence, however, of the irritability of the stomach, but one dose of the medicine was given, and on the following morning at about half past 8 o'clock, we were summoned in great haste to visit him, and found him on his back, breathing with great energy and rapidity, with his face greatly flushed, and the peculiar appearance of his face and eyes, noticed yesterday, were more marked. He had a negro man lying across his knees, and was calling incessantly for the application of cold water to his head, and begging to be bled. On examining the pulse we found it 120 in the minute, large and strong. His whole muscular system was slightly convulsed every few minutes, and this incessant spasmodic action was so violent in his legs that he kept the negro man lying across his knees to prevent the sudden and painful flexion, to which they were liable every moment. In about half an hour after we arrived his entire system became violently convulsed, every muscle seeming to be affected. The contents of the stomach were spasmodically ejected, and the entire body kept in convulsive agitation as if by successive discharges of a powerful galvanic battery. He continued in this condition for some minutes, and was calling incessantly during the whole time for water to his head and for the lancet, until the muscles of the glottis became implicated, respiration suspended, and entire relaxation of the system, with cessation of the pulse at the wrist, put an end to the paroxysm. On recovering from this state of relaxation, which he did in a few minutes, the pulse resumed its volume and frequency, and the impulse of the heart its strength, the face its flushed condition, and the muscular system became again affected as described on our entrance into the room. When interrogated he complains of no pain except that already refered to in the region of the occiput, and the sense of tension through the temples. Sixteen leeches were now applied to his temples, and warm water applied for several hours to encourage the bleeding, but notwithstanding this depletion there was a recurrence of another paroxysm similar to the one just described; making the third in the course of the forenoon: one before we saw him, and two whilst we remained with him. After the third paroxysm he was left in a state of extreme nervous impressibility, the slightest noise or motion of the bedclothes producing spasmodic movements of the muscles. During the remainder of the day he insisted upon having cold water applied to his head, and kept the negro boy lying across his knees; was restless and anxious from apprehension of a return of the convulsions. At bedtime blisters were applied to the top of the sternum and to the inner sides of the calves of the legs, and he remained throughout the night comparatively quiet, though prevented from sleeping by the constant twitching of the muscles and perhaps by *mental* excitement.

On visiting him the next morning we found him lying on his right side coiled up in bed with the bedding carefully tucked under his body, with hot bricks to his feet, and the cold application to his head. He could with difficulty be induced to change his position in bed for a moment at a time, and when the bedclothes were at all disturbed by his change of position, he would call upon his attendants to tuck them under, his commands being in these terms—"tuck me, tuck me," and this call was made incessantly through the night, and was being made every moment during the time we remained with him, and yet on questioning him we could not get him to admit that he felt at all cold or chilly, nor could we elicit from him any reason for his obstinate persistence in lying on his right side. The peculiar frown, the wild and staring eye, the reluctant but hurried answers, the muscular twitchings, the quick perception of whatever was said or done in the room, the constant call to be 'tucked,' the demand to be bled, and to have cold to his head, thirst, uneasiness in the occipital region, sense of tension through the temples, slight nausea, with a pulse 108, large and strong, and violent impulse of the heart, with uniform warmth of the surface, were the noticeable symptoms at this visit. We also now for the first time had our attention particularly drawn to obstinate priapismus which he said had been annoying him for several days. We ordered twenty leeches to the mastoid region, and the bleeding to be encouraged by the application of warm water, and his bowels to be moved by enema. This treatment seemed to make no decided impression on the symptoms, the uneasiness in the occipital region and the priapismus continuing. Calomel and opium were given with the view of procuring sleep, and ptyalizing, but all the symptoms remained without material modification, except the pulse which exhibited repeated sudden changes in frequency and force, until the morning of ——,* when there was a return of the convulsons. This time the convulsions were more violent than before, but in all essential respects identical with those described above. There were several recurrences of them during the day, and in several of them the suspension of respiration was so prolonged that dissolution was apprehended. After he had completely rallied from the last one, we determined to bleed him, and accordingly drew from his arm a pint of blood, with the effect of reducing the heart's action and producing free perspiration. In the course of an hour after this bleeding there were indications of another convulsion, and his arm was untied and blood allowed to flow to the amount of another pint, so that he lost in all about thirty-two ounces of blood. After this depletion he became much more calm, the muscular twitchings became less troublesome, but the other symptoms continued.

He had not slept more than an hour at a time since the first visit with Dr. Bullitt, and his sleep had been disturbed by startings and muscular spasm to such an extent that it was doubtful whether he had been at all relieved by it. It was therefore determined to procure sleep if possible, and also to bring him under the influence of mercury. With this view he was ordered small doses of calomel through the day, and at night morphine

* Near three weeks from the time he was taken home, but the exact date not remembered.

in grain doses every two hours until he slept. He took in the course of the night five grains of morphine, with no other effect than that of rendering him less restless. For several successive nights the effort to induce sleep was repeated, and the calomel continued during the day. Laudanum was used by the rectum and by the mouth without any decided effect. In a few days his stomach became so irritable that the calomel had to be discontinued. His bowels were moved whenever it was thought necessary without difficulty, either by injections or by mild cathartics administered by the mouth, and the discharges were always natural in appearance. Urine continued to be voided in large quantities, but the symptoms first noticed remained without modification.

His strength was such that he could get up to stool without assistance, and notwithstanding the depletion, his face was still considerably flushed. His appetite was capricious, sometimes craving strong food, and again revolting at everything in the shape of nourishment, but his stomach did not at any time retain anything stronger than tea, and it was remarked that animal broths or jellies produced an increase of arterial excitement.

Soon after he was removed home, we noticed great capriciousness of conduct, and occasional wandering, when there was nothing to excite his attention. At one time he would not allow any one but his sisters to be near him, and then, without cause, he would abruptly order them from the room, and request the attentions of his father, who, in his turn, would be sent off, and a negro man, who assisted in nursing, called. This disposition to change his attendants was constantly manifested, and he never could be induced to give a reason for it, but when reminded of his strange conduct, would express the apprehension that he was becoming idiotic. Finally, in the course of a few days after the last attack of convulsions, he became decidedly delirious. At first his delirium was such that he could be made to understand his situation, and after continuing several days passed off, and left him in the condition in which he was previous to its occurrence. But after a short lucid interval it returned in so marked a form that he could not be reasoned with at all. This delirium continued with occasional lucid intervals until within a few days of his death, when he became more rational, so that we could control his mind whenever his attention was excited. During the whole of this time he did not sleep soundly more than once, and that was for about six hours, after taking large doses of digitalis and hyosciamus.

It would be useless to detail the treatment from day to day; indeed it would be impossible, as no notes of the case were taken at the time. Sufficient, that bleeding, both general and local, tartar emetic, digitalis, opiates, hyosciamus, blisters, etc., etc., were tried without the slightest favorable effect. Indeed we were in doubt whether any of these medicines produced any of their appropriate effects. Some of them were used in extraordinary doses without the slightest effect.

He expired on the 6th of January, about five weeks after the supervention of convulsions, and three months after he was arrested.

Inspection of the brain forty-eight hours after death disclosed the following appearances: Very firm and extensive adhesion of the dura mater to the inner table of the cranium. Distinct opacity of the cerebral portion of the arachnoid on the upper surface of the hemispheres, and over the cerebellum. Effusion of serum into the cells of the pia mater generally. Immediately around the verriform process of cerebellum limited effusion of lymph. Extreme vascular engorgement of the membranes covering the cerebellum and medulla oblongata.

On cutting away the cerebral substance from above downwards, it was found of ordinary firmness until we reached the corpora striata and optic thalami, in both of which there was evident softening. The left optic thalamus was of the consistence of thick cream, the corpora striata and right thalamus about that of cerebral matter in the incipient stages of decomposition. Effusion into the lateral ventricles of a straw colored liquid of the consistence of synovial fluid, to the amount of about two drachms in each ventricle. The fourth ventricle dilated to about four times its natural capacity. The fluid which it contained had escaped in removing the brain, and remained in the occipital fossæ in part. It was similar in character to that found in the lateral ventricles, and was supposed to be about half an ounce in quantity.

In a legal point of view this case is deeply interesting; and the conclusion to which we have arrived is, that the strange aberrations of conduct to which he was liable, resulted from undue mental excitement dependent upon a pathological condition of the brain. We arrived at this conclusion before the convulsions supervened, and if a lingering doubt had remained, every vestige of it would have been removed, by the subsequent history of the case, and by the appearances which the post-mortem examination revealed. In this view of the case we are further confirmed by the concurrence of various physicians of respectability, who witnessed the progress of the case, and became acquainted with its history, and still further by others who came to the same conclusion by simply observing his countenance, and the expression of his eyes.

To what extent such a person should be held responsible for his acts, is a question of deep interest to the philanthropist. It seems to us, that his conduct being the result of disease of the brain, *or of that species of moral insanity denominated "undue mental excitement,"* he should have been subjected to no harsher treatment than that of confinement in an asylum for the insane. The severe penalty which the law imposes for the act for which he was imprisoned, could not have been applied in his case by any enlightened and christian jury.

March 12, 1849.

